# Systematic reviews of clinical laboratory studies: Pilot risk of bias tool developed by consensus

**DOI:** 10.1002/cesm.12098

**Published:** 2024-08-12

**Authors:** Tilly Fox, Beverley J. Hunt, Robert A. S. Ariens, Greg J. Towers, Robert Lever, Paul Garner, Rebecca Kuehn

**Affiliations:** ^1^ Department of Clinical Sciences Liverpool School of Tropical Medicine Liverpool UK; ^2^ King's Healthcare Partners London UK; ^3^ Leeds Institute of Cardiovascular and Metabolic Medicine University of Leeds Leeds UK; ^4^ Division of Infection and Immunity University College London London UK; ^5^ Imperial College London NHS Trust London UK

**Keywords:** clinical laboratory, COVID‐19 pathophysiology, critical appraisal, post‐COVID‐19 condition, risk of bias, systematic review, validity assessment

## Abstract

**Introduction:**

Some research studies aim to elucidate pathophysiology by examining blood or tissue markers in relation to clinical findings. In COVID‐19, this has led specialists to promote treatment options based on single studies without systematic appraisal and critical summaries of the data. As we could not identify any published tools for this purpose, we developed a pilot risk of bias tool by consensus, and report here on our approach.

**Methods:**

Using an expert consultative consensus process, a panel of five topic experts were guided through a set of iterative steps to develop questions intended to elicit information about the study methods and reporting in clinical laboratory studies. The team piloted the tool in three clinical laboratory studies, and then applied it formally as a component in assessing a hypothesis about mechanisms in the post‐COVID‐19 condition as part of a Cochrane review.

**Results:**

The pilot tool assessed study quality and bias across three domains applicable to comparative and single‐arm clinical laboratory studies: collection and handling of samples, experimental methods, and reporting of the results. In the Cochrane review, the tool identified substantive risk of bias in the included clinical laboratory studies.

**Conclusion:**

The plethora of COVID‐19 research has highlighted the need for formal methods to systematically appraise clinical laboratory studies related to disease pathology. This tool provides a systematic approach to appraise the validity of these studies. Our process may guide others in the development of appraisal tools in areas where they are needed. Given the relationship between clinical laboratory studies and the development of medical treatments, further development of this risk of bias tool is important for evidence‐based healthcare and research.

## 1 INTRODUCTION

Systematic review methods are being applied more widely beyond randomized controlled trials (RCTs), and developments to systematic review methods have allowed critical appraisal of evidence from non‐randomized study designs [1]. Mechanistic study designs and in vitro studies, among other sources of evidence, lack methods for assessments of validity within evidence syntheses. This methodological gap has been identified in assessments of environmental health hazards where systematic reviews often seek evidence from experimental animal data, observational reports, and studies of molecular events [2]. Similarly, in COVID‐19, vast quantities of biomedical research into radiologic, virologic, tissue changes, and blood markers have been used to identify putative disease mechanisms, relating these to clinical findings. These are sometimes used in the pathway to identify treatments; however, the methodological quality of some studies has been questioned [3].

Our review development team at the Cochrane Infectious Diseases Group experienced the paucity of risk of bias tools for alternative evidence sources when planning a review on “Plasmapheresis to remove amyloid fibrin(ogen) particles for treating the post‐COVID‐19 condition” [4]. The review sought to appraise evidence from clinical laboratory studies that provided the rationale for plasmapheresis as a treatment for removing amyloid fibrin(ogen) particles in people with post‐COVID‐19 condition. The review motivation and the justification for inclusion of this evidence stream is detailed in Box 1. This paper describes the initial consultative expert consensus process used for the development of a pilot tool to assess bias in clinical laboratory studies.

Box 1: **Why do we need to appraise clinical laboratory studies?**

A team led by Cochrane Infectious Diseases Group became aware that some people with post‐COVID‐19 condition (PCC) are undergoing apheresis aiming to remove so‐called “microclots” from their vascular system [5]. A Cochrane systematic review was conducted to appraise the evidence for this treatment [4]. The preliminary evaluation revealed no trials on this topic. Using an iterative process we then turned to examining evidence from other study designs relevant to this treatment for the post‐COVID‐19 condition.

We identified laboratory studies that investigated the theory that “microclots” contribute to symptoms associated with post‐COVID‐19 condition. These studies utilized fluorescence microscopy and flow cytometry to investigate the presence of amyloid fibrin(ogen) particles in plasma from participants with post‐COVID‐19 condition, and in some cases to compare this with controls. These studies were used to support apheresis treatments to remove amyloid fibrin(ogen) particles in people with post‐COVID‐19 condition without any formal appraisal of the validity of this evidence. We argue that it is important to apply critical appraisal methods, drawing on the Cochrane risk of bias approach, to evaluate the studies that had been conducted and used to justify a hypothesis that apheresis might be effective as treatment for PCC.

A consultative expert consensus process was identified as the most robust tool development method within our context of immediate need. Further details of this process, including the strengths, limitations, and suggested work required to further validate this tool are outlined in the discussion.

## 2 MATERIALS AND METHODS

The research team (TF, RK) initially searched the LATITUDES network [5] and contacted experts in the field of systematic review methods to identify appropriate risk of bias tools for this study design. No risk of bias tools specific to the appraisal of clinical laboratory studies were identified. We therefore planned to utilize a consultative expert consensus panel approach to develop a pilot risk of bias tool appropriate to the appraisal of these study designs for application within our systematic review. The consultative expert consensus method involves consultation of selected individuals with topic area expertise to gain their ideas and perceptions on the topic. Central team members (in our case, a chair and co‐chair) then utilize this information to develop the product (pilot risk of bias tool for clinical laboratory studies), before gaining further feedback from the expert panel to refine this and reach consensus among the team [6].

Our consultative expert consensus discussion calls were held via Zoom, with the agenda and methods set out by the research team. To generate ideas and achieve consensus, a nominal group technique was used [7]. We defined consensus as all panel members reaching agreement, which was feasible due to the small panel size. We found that it wasn't necessary to hold votes on the ideas proposed, as consensus could be reached through discussion. The final decisions that had been developed through consensus were presented to the panel at the end of each call by the researchers to ensure that the appropriate responses were captured. Whilst the researchers were involved in the discussions, they did not contribute to final consensus decisions.

The advantage of this method is that the consultation allows access to expertise from highly qualified individuals to support the decision‐making process, and the use of consensus ensures that all contributors are satisfied with the end‐result to prevent unbalanced influence from one party [7]. The disadvantage compared to other methods such as the Delphi process is that participating individuals are selected, and there is no open call to other individuals who may wish to participate. This means key perspectives may not be captured, which is inflated by the small number of individuals that were included. Due to the immediate need for the systematic review (detailed in the introduction of the review), we were restricted by a limited timeframe to produce this pilot risk of bias tool, hence we selected a small consultative expert consensus as the best‐fit methodology. Based on this, we refer to this as a “pilot” tool with scope for further validation.

We mapped our procedures against the recently published ACcurate COnsensus Reporting Document (ACCORD) in Appendix 1 [8]. We did not register this tool development process but prospectively registered the Cochrane review for which the tool was initially developed on 24 February 2023 [9].

**Stage 1: Expert panel.**

We assembled a panel of five experts around the review topic to contribute to consensus discussions for pilot tool development. Two researchers (TF, RK) with evidence synthesis expertise acted as chair and co‐chair to identify panel members, facilitate the consensus discussions, and provide materials for review by the expert panel.

The individuals on the expert panel were identified through our contacts and recommended as individuals with expertise in laboratory methods, translational thrombosis and haemostasis, and evidence synthesis. Restricted by our limited timeframe, we rationalized that it was reasonable to include two individuals with expertise in each topic of laboratory methods and translational thrombosis in COVID‐19 to attempt to balance opinions from these perspectives. On top of this, we included one individual with evidence synthesis expertise who had also experienced post‐COVID‐19 condition.

The research team initially approached each individual via email between September and October 2022 to discuss the rationale of this project and confirm their attendance on the panel; all five individuals agreed to participate and contributed to all stages of the tool development process. The expertise of all individuals involved in the pilot tool development is described in Table 1.

**Table 1:** Expertise and roles of individuals involved in development of a pilot tool for assessment of bias in clinical laboratory studies.

Name	Expertise	Role
Tilly Fox	Evidence synthesis methodology and production	Project chair. Identification of panel members, coordination of consultative consensus process, production of draft tool, pilot and validation of tool.
Dr. Rebecca Kuehn	Evidence synthesis methodology and production	Project co‐chair. Identification of panel members, support of consultative consensus process, production of draft tool, pilot and validation of tool.
Professor Beverley J. Hunt	Clinical and translational thrombosis	Consensus panel member
Professor Robert A. S. Ariens	Clinical and translational thrombosis	Consensus panel member
Professor Greg J. Towers	Laboratory methods	Consensus panel member
Dr. Robert Lever	Laboratory methods	Consensus panel member
Professor Emeritus Paul Garner	Evidence synthesis methodology and patient perspective of post‐COVID‐19 condition	Consensus panel member

**Stage 2: Consensus round 1 to develop domains.**

The objective of the first consensus round was to reach agreement on the domains of bias that should be addressed in the tool. This consensus round took place in November 2022.

The researchers searched for existing risk of bias tools via the LATITUDES network and analysed these to determine a planned structure for our new tool. The Cochrane risk of bias 2 (RoB2) tool and the Cochrane risk of bias in non‐randomized studies of interventions (ROBINS‐I) tool uses a logical structure to assess bias with high uptake, so we decided to base our tool on the same structure. This includes the use of “domains” (mechanisms by which bias can be introduced) and “signaling questions” (a series of questions that aim to elicit information about features of the study that are relevant to risk of bias) [10, 11].

These tools were sent to the panel before the first consensus Zoom discussion to allow them an opportunity to familiarize themselves with the structure. In the first consensus round, the panel mapped the applicability of the domains in these two tools to the appraisal of clinical laboratory studies and discussed whether these mechanisms of bias were relevant to this study design. They also discussed whether any other mechanisms of bias were relevant to laboratory studies that should be included as a domain in the tool.

From this exercise, the consensus panel agreed on three domains to assess bias in clinical laboratory studies: collection and handling of samples, experimental methods, and reporting of the results. The results of the mapping activity are presented in Appendix 2.

**Stage 3: Consensus round 2 to develop signaling questions.**

The second set of consensus rounds concerned the development of signaling questions relevant to the appraisal of clinical laboratory studies. In similarity to the RoB2 tool, this tool was designed to be applied at the outcome level, so questions were primarily targeted at individual outcomes.

The objective of this consensus discussion was to curate simple questions that can be used to validate clinical laboratory methods and determine whether steps have been taken within these studies to minimize bias. The research team conducted a series of scoping searches on PubMed to seek evidence on biases in laboratory studies, using combinations of the terms “laboratory,” “experiment,” “bias,” and “validity.” We identified very minimal published evidence on biases in laboratory studies to support this process but were able to refer to literature examining bias in in vivo animal studies and draw similarities between biases in these study designs [12, 13]. The research team shared relevant literature with the panel members via email before the consensus round.

During the consensus rounds, the panel members worked through each of the curated domains to discuss important questions that could be used to elicit the introduction of bias into laboratory studies. These discussions were based on their individual knowledge and the relevant literature. Three rounds of discussion were required to work through all three domains and reach consensus on the final signaling questions, and panel members contributed to all discussions. These consensus discussions were held in November and December 2022.

The questions were designed to be applied to controlled clinical laboratory studies, however, we realized that it would be useful if this tool could be applied to uncontrolled studies and that questions relating to control groups could be omitted when used to assess bias in uncontrolled studies. We also designed the questions to be answered as yes/no/no information and intended that the answers for all signaling questions within one domain should be combined to provide an overall judgement regarding the bias present in that domain. This can be judged as low, some concerns, or high risk of bias. These methods are based on the RoB2 and ROBINS‐I tools [10, 11].

The research team used these signaling questions to build a draft risk of bias tool and recorded the rationale behind the domains and signaling questions developed.

**Stage 4: Pilot**

This stage tested the usefulness of the tool and evaluated whether it could be used to assess bias across a range of clinical laboratory studies. To do this, the researchers piloted the tool using four published studies. These studies were randomly selected using methods described in Appendix 3. Two researchers (TF and RK) independently applied the tool to these studies.

Despite the different laboratory techniques used and outcomes being assessed, the tool allowed a systematic assessment of bias across outcomes in all four studies (see Appendix 3 for results of pilot), and we felt that it was straightforward to apply and addressed all important biases. This pilot suggested that this may be a useful tool to assess bias across various clinical laboratory scenarios.

The researchers fed back their experiences of the application of this tool to the panel via email, and no suggested changes were made.

**Stage 5: Final consensus**

The researchers provided the panel with a final opportunity to review the tool and give additional feedback via email. We did not receive any suggested changes to the tool.

**Stage 6: Application and peer review**

This tool was applied in the Cochrane review to assess bias in five clinical laboratory studies, where it was peer‐reviewed at protocol and final review stage of the Cochrane review [4]. We implemented minor suggestions to improve the clarity of language in the questions.

## 3 RESULTS: A PILOT TOOL TO ASSESS BIAS IN CLINICAL LABORATORY STUDIES

The rationale and purpose of each domain included in the tool is detailed below.

**Domain 1: Collection and handling of samples**

Domain 1 assesses the collection and handling of samples and aims to identify whether there are any concerns related to how participants were identified, how samples were obtained, and how bias between study groups has been minimized. Ensuring that the collection and handling of samples has been conducted in a systematic and well‐planned manner, and that all samples have been handled in the same way, reduces confounding at this stage.

**Domain 2: Experimental methods.**

Domain 2 attends to the experimental methods used. This domain is based on domain 4 from the RoB 2 tool ‘Risk of bias in measurement of the outcome’, which aims to determine if the methods used to measure the outcome are appropriate and that bias has been minimized throughout the experiments themselves. We would expect validated methods to be used and robust statistical analyses to be planned to compare results between populations of interest.

**Domain 3: Reporting of results.**

Domain 3 relates to the reporting of the results and is based on domain 5 of the RoB 2 tool. This domain assesses whether the results have been appropriately quantified and analyzed, ensuring that all results are included in the analysis and the result is not influenced by missing data.

This pilot tool is intended to be used in systematic reviews where it is necessary to appraise the methods of clinical laboratory studies, such as those used to compare clinical parameters between two or more groups. The application of this tool within our Cochrane review is outlined in Box 2.

Box 2: **Application of the tool in a Cochrane Systematic Review**

In our Cochrane review the tool was used to appraise five laboratory studies, four of which utilized fluorescence microscopy [14, 15, 16, 17] and one that used flow cytometry [18] to evidence the presence of amyloid fibrin(ogen) particles in platelet‐poor plasma (PPP) samples from people with post‐COVID‐19 condition. In three studies, samples from a control group were also investigated [14, 16, 18].

The pilot risk of bias tool allowed us to identify issues across the five studies, including consistent concerns relating to the source of participants, the methodology used, and the lack of comparison between matched samples from the PCC and control groups. The full report of our risk of bias assessment is available in Appendix 1 of the Cochrane review [4] and concludes that four of these studies are at high risk of bias, and there are some concerns relating to the final study.

Our risk of bias assessment allowed us to understand that the conclusions drawn by the laboratory studies, in terms of the association between amyloid fibrin(ogen) particles and post‐COVID‐19 condition, are not sufficiently evidenced by the studies. Ultimately, we were able to conclude that there is no rationale for plasmapheresis to remove amyloid fibrin(ogen) particles in PCC, and this treatment should not be received outside of the context of an RCT.

The full tool is presented in Table 2, formatted in a similar style to the RoB2 tool. We have provided an explanation of the purpose of each signaling question to aid users in how to interpret and answer the questions. The tool is intended to be applied at the outcome level, meaning users should use the tool to create a risk of bias judgement for each critical outcome reported in the study. Users can apply the tool to assess bias in both controlled and uncontrolled clinical laboratory studies, but questions that refer to a control group should be omitted in the case of assessing uncontrolled study designs. An example of the application of the tool to uncontrolled studies is available in the Cochrane review [4].

**Table 2:** Pilot risk of bias tool to appraise clinical laboratory studies.

**Domain 1: Collection and handling of samples**		
Have patients been clinically evaluated to ensure they fulfil the criteria for inclusion?	This question is used to ensure that appropriate diagnostic tests been performed to ensure that participants providing samples meet the criteria for the exposure and control groups. Samples taken from participants who have not been formally diagnosed may not accurately represent the population group.	Yes/No/No information
Have statistical calculations been performed to determine an appropriate sample size?^a^	This is required to ensure the sample size is large enough to detect a difference between the groups. Underpowered studies may yield significant results more easily and lead to the publishing of experiments that were never necessarily intended to be published.	Yes/No/No information
Are samples contemporaneous?	The integrity of samples obtained at different time points will be affected by the time spent in storage.	Yes/No/No information
Have patients been matched on non‐investigational characteristics that may introduce confounding?	Matching characteristics across groups that are not being investigated, such as age, sex, and health status, reduces confounding bias.	Yes/No/No information
Have samples from both groups been collected and prepared in the same way?	Samples should be consistently handled to ensure this does not influence how they respond in laboratory experiments, e.g., test and control samples should be subjected to the same number of freeze‐thaw cycles and sampling to avoid biased degradation or contamination.	Yes/No/No information
**Risk of bias**	If all above questions are answered “yes,” there is a low risk of bias. If one question is answered “no” or “no information” there are some concerns. If more than one question is answered “no” or “no information” there is a high risk of bias for this domain.	Low/Some concerns/High
**Domain 2: Experimental methods**		
Is a validated methodology used? If not, is the methodology well described?	Methods used may be widely accepted, or new methods can be used that are adequately described and justified.	Yes/No/No information
Has a normal range been calculated?	This study design requires appropriate statistical calculations to establish a normal range (typically considered a range of values that you would expect to see for 95% of a healthy population when conducting a specific test).	Yes/No/No information
Have statistical methods been planned to compare the values of experimental groups with controls?	Prespecified methods for comparing the values of experimental groups with controls ensures that a statistical plan is followed.	Yes/No/No information
Are the people evaluating the data blind to the source of the samples?	Knowledge of the group from which the samples have been obtained may introduce bias in evaluation of the data.	Yes/No/No information
Is an internal and external control used?	An internal control is used to monitor and assure the reproducibility of results. An external control is a real, positive clinical sample used to monitor for errors in the experimental process.	Yes/No/No information
**Risk of bias**	If all above questions are answered “yes,” there is a low risk of bias. If one question is answered “no” or “no information” there are some concerns. If more than one question is answered “no” or “no information” there is a high risk of bias for this domain.	Low/Some concerns/High
**Domain 3: Reporting of the results**		
Are the results quantified and with appropriate analysis?	Results should be fully quantified to allow appropriate analysis.	Yes/No/No information
Are all, or nearly all, data available?	An appropriate analysis should include data from all samples that were included.	Yes/No/No information
Are appropriate statistical tests used to test for differences between groups?	This allows validated comparison between values.	Yes/No/No information
Are the data available in a repository and accessible to others?	Laboratory data should be made publicly available to allow external analysis and comparison. Coefficients of inter and intra assay variation should be made available.	Yes/No/No information
**Risk of bias**	If all above questions are answered “yes,” there is a low risk of bias. If one question is answered “no” or “no information” there are some concerns. If more than one question is answered “no” or “no information” there is a high risk of bias for this domain.	**Low/Some concerns/High**
**Overall risk of bias**	The overall risk of bias is calculated based on the highest domain judgement. If there is high risk of bias in one or more domains, the overall risk of bias is high. If all domains are at low risk of bias, the study is at overall low risk of bias for this outcome.	**Low/Some concerns/High**

a This signaling question was added in November 2023 after the development of the original tool, following a discussion with the panel.

## 4 DISCUSSION

This tool to assess risk of bias in clinical laboratory studies allows a variety of stakeholders to assess the validity of evidence to understand disease pathophysiology and hence to underpin possible treatment options (Table 3). The ability to formally appraise the robustness of studies of pathophysiology will be increasingly important as people seek to critically evaluate evidence related to the biological mechanisms driving many diseases including COVID‐19 and post‐COVID‐19 pathogenesis. This tool will be valuable to these investigations and may enable researchers to identify potential treatments with stronger evidential basis worthy of further rigorous evaluation. We expect that the tool may also be informative for clinical laboratory studies in other disease areas, including cancer, cardiovascular, endocrinology, and rare diseases.

**Table 3:** The usefulness of the pilot risk of bias tool for different stakeholders.

Stakeholder	Purpose of the tool
Laboratory scientists	Guides individuals in understanding which aspects of study methods and reporting will be appraised in the interpretation of their work.
Clinical trial developers	Supports decision‐making for determining if there is a reliable evidential basis from clinical laboratory studies to develop further clinical studies.
Clinicians	Provides guidance for how to interpret the findings of clinical laboratory studies to make informed healthcare decisions.
Systematic reviewers	Allows the inclusion and appraisal of clinical laboratory studies which may be a valuable source of evidence. Demonstrates a systematic approach for developing risk of bias tools within the constraints of time‐dependent review production.
Decision‐makers	Facilitates the interpretation of alternative sources of evidence which may contribute to decision‐making.

Whilst clinical laboratory studies are so far rarely appraised within systematic reviews, there are instances where their appraisal is fundamental to the production of high‐priority evidence syntheses, and having access to a robust tool to support these appraisals is important.

### 4.1 Strengths and limitations

This tool is the first of its kind designed specifically to allow assessments of bias in clinical laboratory studies, the rationale for which has been discussed throughout this article. The choice of a consultative expert consensus process to develop this tool allowed us to prepare it rapidly and facilitated in‐depth discussions around the sources of risk of bias from people familiar with the laboratory methods.

Through our pilot stage and the Cochrane systematic review, we applied this tool to a total of eight laboratory studies that cover a variety of clinical topics and adopted a variety of methods, which demonstrates the flexibility of the tool to assess bias across heterogenous studies. The usefulness of the tool in systematic reviews has been demonstrated through our previous publication, where the tool enabled us to conclude that the laboratory studies assessed were not reliable evidence.

Despite the clear utility of such a tool, we recognize the trade‐off between a consultative expert consensus panel with a small number of invited participants and widespread use across different topics. The small panel may reflect limited expertise in comparison to a larger panel, and the lack of open invitation for participants means that individuals with important expertise did not have an opportunity to contribute. As a result, other important sources of bias in clinical laboratory studies may not have been captured. Nevertheless we produced a bespoke instrument rapidly and fit for purpose.

The pilot stage of the tool development process attempted to identify any obvious limitations within the tool itself, however, it was applied to a small number of studies and some issues with its application may not have been identified. A specific concern relates to domain 2 of this pilot tool, which focuses on the assessment of bias in the experimental methods. Differences in the experimental methods used across clinical laboratory studies may mean that this domain requires study‐specific adaptations. For example, to reach consensus on how a ‘validated assay’ is defined. Feedback from users of the tool will be valuable to ascertain whether adaptation is needed.

Beyond this, our pilot process was limited to the application of this tool in a systematic review setting, and we do not know if this will be adopted by the other stakeholders outlined in Table 3.

## 5 FUTURE WORK

Further application and validation of the tool by external researchers in other settings beyond post‐COVID‐19 condition will be important to continue to develop this tool and understand its applicability in different scenarios. There may be a rationale to utilize a Delphi consensus process with a larger number of contributing experts to produce a second iteration of this tool, as was used in the development of the RoB2 and ROBINS‐I tools [19]. We invite stakeholders, such as those outlined in Table 3, to test the usefulness of this tool in practice and provide feedback on its use. Comments and feedback can be provided to tilly.fox@lstmed.ac.uk and will be utilized to further develop this tool. Experts with interest in contributing to future Delphi consensus for this tool are also encouraged to contact the author team.

## CONCLUSION

This paper describes the development of a new pilot risk of bias tool that we expect to be a valuable instrument for researchers to appraise evidence from clinical laboratory studies which aim to demonstrate a relationship between clinical characteristics and laboratory parameters.

## AUTHOR CONTRIBUTIONS

**Beverley J. Hunt**: Investigation; writing—review and editing. **Robert A. S. Ariens**: Investigation; writing—review and editing. **Greg J. Towers**: Investigation; writing—review and editing. **Robert Lever**: Investigation; writing—review and editing. **Paul Garner**: Conceptualization; investigation; methodology; writing—review and editing. **Rebecca Kuehn**: Conceptualization; data curation; formal analysis; investigation; methodology; validation; writing—review and editing.

## CONFLICT OF INTEREST STATEMENT

The authors declare no conflict of interest.

## PEER REVIEW

The peer review history for this article is available at https://www.webofscience.com/api/gateway/wos/peer-review/10.1002/cesm.12098.

## ETHICS STATEMENT

The authors have nothing to report.

## Data Availability

Data sharing is not applicable to this article as no datasets were generated or analysed during the current study.

## APPENDIX 1

See Table A1.

**Table A1:** ACcurate COnsensus Reporting Document (ACCORD) mapping.

Item No.	Section	Checklist Item (*help text*)	Detail (page number)
T1	**Title**	Identify the article as reporting a consensus exercise and state the consensus methods used in the title. *For example, Delphi or nominal group technique.*	The title reflects the use of consensus methods [1].
I1	**Introduction**	Explain why a consensus exercise was chosen over other approaches.	See introduction and methods for justification of consultative expert consensus [4].
I2		State the aim of the consensus exercise, including its intended audience and geographical scope (national, regional, global).	Scope of the consensus exercise is outlined in the Methods [17]
I3		If the consensus exercise is an update of an existing document, state why an update is needed, and provide the citation for the original document.	N/A
M1	**Methods**	If the study or study protocol was prospectively registered, state the registration platform and provide a link. If the exercise was not registered, this should be stated. *Recommended to include the date of registration.*	Tool development was not prospectively registered [17].
M2	**Selection of SC and/or panellists**	Describe the role(s) and areas of expertise or experience of those directing the consensus exercise. *For example, whether the project was led by a chair, co‐chairs or a steering committee, and, if so, how they were chosen. List their names if appropriate, and whether there were any subgroups for individual steps in the process.*	Table 1 [5].
M3		Explain the criteria for panellist inclusion and the rationale for panellist numbers. State who was responsible for panellist selection.	Inclusion criteria and rationale detailed in the Methods [5]
M4		Describe the recruitment process (how panellists were invited to participate). *Include communication/advertisement method(s) and locations, numbers of invitations sent, and whether there was centralised oversight of invitations or if panellists were asked/allowed to suggest other members of the panel.*	Recruitment process outlined in the Methods [5]
M5		Describe the role of any members of the public, patients or carers in the different steps of the study.	Role of individual who has experienced post‐COVID‐19 described [5]
M6	**Preparatory research**	Describe how information was obtained before generating items or other materials used during the consensus exercise. *This might include a literature review, interviews, surveys, or another process.*	Details of scoping searches on PubMed and the LATITUDE network described [6, 7].
M7		Describe any systematic literature search in detail, including the search strategy and dates of search or the citation if published already. *Provide the details suggested by the reporting guideline PRISMA and the related PRISMA‐Search extension.*	N/A No systematic literature search conducted.
M8		Describe how any existing scientific evidence was summarised and if this evidence was provided to the panellists.	Evidence summarized by researchers and presented to panelists before and during consensus discussions [7].
M9	**Assessing consensus**	Describe the methods used and steps taken to gather panellist input and reach consensus (e.g., Delphi, RAND‐UCLA, nominal group technique). *If modifications were made to the method in its original form, provide a detailed explanation of how the method was adjusted and why this was necessary for the purpose of your consensus‐based study.*	Panelist ideas captured during group calls held on Zoom, with modified nominal group technique used [17].
M10		Describe how each question or statement was presented and the response options. State whether panellists were able to or required to explain their responses, and whether they could propose new items. *Where possible, present the questionnaire or list of statements as supplementary material.*	All questions were posed through and responded to during consensus discussions. Responses of panelists were captured through discussion calls [7, 8, 9].
M11		State the objective of each consensus step. *A step could be a consensus meeting, a discussion or interview session, or a Delphi round.*	Objectives of each step clearly stated: Development of domains, development of signaling questions, and final consensus [6, 7, 8].
M12		State the definition of consensus (e.g., number, percentage, or categorical rating, such as “agree” or “strongly agree”) and explain the rationale for that definition.	Consensus defined as all panel members in agreement, which was feasible due to our small panel size [17].
M13		State whether items that met the prespecified definition of consensus were included in any subsequent voting rounds.	N/A
M14		For each step, describe how responses were collected, and whether responses were collected in a group setting or individually.	Responses collected via group call for first two consensus rounds, and final consensus responses collected via email [6, 7, 8].
M15		Describe how responses were processed and/or synthesised. *Include qualitative analyses of free‐text responses (e.g., thematic, content or cluster analysis) and/or quantitative analytical methods, if used.*	N/A, no analytical processes used.
M16		Describe any piloting of the study materials and/or survey instruments. *Include how many individuals piloted the study materials, the rationale for the selection of those individuals, any changes made as a result and whether their responses were used in the calculation of the final consensus. If no pilot was conducted, this should be stated.*	Pilot described in stage 4 [8].
M17		If applicable, describe how feedback was provided to panelists at the end of each consensus step or meeting. *State whether feedback was quantitative (e.g., approval rates per topic/item) and/or qualitative (e.g., comments, or lists of approved items), and whether it was anonymised.*	Final decisions were presented to panelists at the end of each consensus call to ensure the correct responses and decisions had been captured [17].
M18		State whether anonymity was planned in the study design. Explain where and to whom it was applied and what methods were used to guarantee anonymity.	No anonymity was used.
M19		State if the steering committee was involved in the decisions made by the consensus panel. *For example, whether the steering committee or those managing consensus also had voting rights.*	The researchers were involved in discussions but did not contribute to consensus decisions made by the panel [17].
M20	**Participation**	Describe any incentives used to encourage responses or participation in the consensus process. *For example, were invitations to participate reiterated, or were participants reimbursed for their time.*	All invited participants responded to the invitation to participate, and no incentives were used [5].
M21		Describe any adaptations to make the surveys/meetings more accessible. *For example, the languages in which the surveys/meetings were conducted and whether translations or plain language summaries were available*.	N/A All panel members had strong scientific knowledge and no accessibility requests.
R1	**Results**	State when the consensus exercise was conducted. List the date of initiation and the time taken to complete each consensus step, analysis, and any extensions or delays in the analysis.	Dates of consensus discussions and number of rounds stated [6, 7, 8].
R2		Explain any deviations from the study protocol, and why these were necessary. *For example, addition of panel members during the exercise, number of consensus steps, stopping criteria; report the step(s) in which this occurred.*	No published protocol but we did not deviate from the planned methods.
R3		For each step, report quantitative (number of panellists, response rate) and qualitative (relevant socio‐demographics) data to describe the participating panellists.	Panelists participated in all steps [8].
R4		Report the final outcome of the consensus process as qualitative (e.g., aggregated themes from comments) and/or quantitative (e.g., summary statistics, score means, medians and/or ranges) data.	Outcomes of the consensus process presented in Appendix 2, Appendix 3, and Table 2 [12, 13].
R5		List any items or topics that were modified or removed during the consensus process. Include why and when in the process they were modified or removed.	N/A
D1	**Discussion**	Discuss the methodological strengths and limitations of the consensus exercise. *Include factors that may have impacted the decisions (e.g., response rates, representativeness of the panel, potential for feedback during consensus to bias responses, potential impact of any non‐anonymised interactions).*	Strengths and limitations outlined in methods and discussion [5, 17, 18, 20].
D2		Discuss whether the recommendations are consistent with any pre‐existing literature and, if not, propose reasons why this process may have arrived at alternative conclusions.	N/A There are no existing risk of bias tools for laboratory studies.
O1	**Other information**	List any endorsing organisations involved and their role.	N/A
O2		State any potential conflicts of interests, including among those directing the consensus study and panellists. Describe how conflicts of interest were managed.	Authors declare no competing interests [1].
O3		State any funding received and the role of the funder. *Specify, for example, any funder involvement in the study concept/design, participation in the steering committee, conducting the consensus process, funding of any medical writing support. This could be disclosed in the methods or in the relevant transparency section of the manuscript. Where a funder did not play a role in the process or influence the decisions reached, this should be specified.*	Funder did not play a role in the consensus process [1].

## APPENDIX 2

See Table B1.

**Table B1:** Applicability of the domains of the Risk of Bias 2 tool and ROBINS‐I tool for assessing bias in clinical laboratory studies.

Domain from RoB2 and ROBINS‐I tool	Consensus group decision on applicability to assessment of bias in clinical laboratory studies
Bias due to confounding	Applicable; confounding factors may distort the association between the condition of interest and the outcome. Study authors should take steps to minimize this bias.
Bias arising from the randomization process	Not applicable due to lack of randomization; samples are obtained from participants with selected characteristics. Bias may still arise in the process of obtaining participants for sampling if methods differ between groups.
Bias due to deviations from the intended interventions	Not applicable due to noninterventional study design.
Bias in classification of the intervention	Not applicable due to noninterventional study design, but bias could arise from the definition of patient groups, so these should be defined a priori.
Bias in selection of the participants into the study	Applicable; participants should be recruited into the study based on characteristics observed before the start of the study.
Risk of bias due to missing outcome data	Applicable; data from all samples should be presented.
Risk of bias in measurement of the outcome	Applicable; laboratory methods should be validated and not differ between samples.
Risk of bias in selection of the reported result	Applicable; all available data should be appropriately analyzed and presented.

## APPENDIX 3

See Table C1.

**Table C1:** Risk of bias assessment of four clinical laboratory studies.

	Pretorius 2021 [20]	Spinel‐Bejarano 2013 [21]	Koh 2007 [22]	Lawrie 2008 [23]
Study ID	Presence of amyloid fibrin(ogen) particles in platelet‐poor plasma	HLA‐DRB1 alleles frequency	Percentage of cerebrospinal fluid leukocytes that were neutrophils	Levels of tumour‐associated *MIRN155* (*miR‐155*) in serum
Domain 1: Collection and handling of samples				
Have patients been clinically evaluated to ensure they fulfil the criteria for the condition?	No: Self‐identified participants	Yes: Clinical diagnostic criteria clearly stated with medical history and physical diagnosis.	Yes: Diagnostic criteria well‐described.	Yes: Clinical diagnosis information provided in Appendix.
Have statistical calculations been performed to determine an appropriate sample size?*	No	No	No	No
Are samples contemporaneous?	No: “One post‐COVID‐19 participant also previously contributed a healthy sample”.	No: No information.	No: Collected between 2001 and 2005.	No: Taken at diagnosis and analysed retrospectively.
Have patients been matched on age, sex, or health status?	No: No statistical comparison of study groups	No: age not applicable but other characteristics not matched across groups.	Yes: No difference in demographic features between groups.	Yes: No difference in demographic features between groups.
Have samples from both groups been collected and prepared in the same way?	No: No evidence of quality control e.g., no evidence of coefficients of variation	Yes	Yes	Yes
Risk of bias for domain 1	High risk of bias	High risk of bias	High risk of bias	High risk of bias
Domain 2: Experimental methods				
Is a validated methodology used? If not, is the methodology well described?	No: The staining methodology is not well described or validated.	No: Methodology for HLA‐DRB1 alleles frequency not well described.	No: No information.	Yes: Methodology well described.
Has a normal range been established in the study or referenced?	No: No reference to normal range.	Yes: OR and 95% CI presented for control group.	Yes: Mean and range presented for control group.	Yes: Median and IQR presented for control group.
Has a statistical test been used to compare values of patients with controls?	No: No statistical tests reported comparing to controls.	Yes: Student t‐test, ANOVA, and nonparametric tests were used.	Yes: Independent sample t‐test.	Yes: Expression levels were compared using Mann–Whitney independent *t*‐test
Are the people evaluating the data blind to the source of the samples?	No: No information	No: No information.	No: No information.	No: No information
Is an internal control used?	No: No information	No: No information.	No: No information.	Yes: As recommended by manufacturer.
Risk of bias for domain 2	High risk of bias	High risk of bias	High risk of bias	Some concerns
Domain 3: Reporting of the results				
Are the results quantified and with statistical analysis? For example mean or median, range or SD.	No: Results are narratively described and no statistical analysis.	Yes	Yes	Yes
Are all, or nearly all, data available? If a subset of data is presented in the article, is this randomly selected?	No: A subset of data is presented in photographs. Does not seem to be randomly selected.	Yes	Yes	Yes
Are appropriate statistical tests used to test for differences?	No: No quantification of data and no statistical tests.	Yes	Yes	Yes
Are the data available on a data repository and available to others?	Yes: Microscopic images available online but without a scale bar. Online statistics and proteomics files inaccessible.	No	No	No
Risk of bias for domain 3	High risk of bias	Some concerns	Some concerns	Some concerns
Overall risk of bias				
	High risk of bias	High risk of bias	High risk of bias	High risk of bias

We piloted our tool on four clinical laboratory studies. The first study [20] was identified in our search for our Cochrane review and the following three studies [21, 22, 23] were selected from a search performed on Google Scholar using the term “Comparative Clinical Laboratory Study,” restricted to studies published after 1 January 2000.
